# Serine/threonine kinase 17b (STK17B) signalling regulates Purkinje cell dendritic development and is altered in multiple spinocerebellar ataxias

**DOI:** 10.1111/ejn.15465

**Published:** 2021-09-29

**Authors:** Qin‐Wei Wu, Josef P. Kapfhammer

**Affiliations:** ^1^ Institute of Anatomy, Department of Biomedicine University of Basel Basel Switzerland

**Keywords:** cerebellar Purkinje cells, DRAK2, PKCγ, spinocerebellar ataxias, STK17B

## Abstract

Serine/threonine kinase 17b (STK17B, also known as DRAK2) is known to be a downstream effector of protein kinase C (PKC) in the immune system, in particular T lymphocytes. PKC activity also plays a critical role for dendritic development and synaptic maturation and plasticity in cerebellar Purkinje cells. We present evidence that STK17B is strongly expressed in mouse cerebellar Purkinje cells starting in the early postnatal period and remaining highly expressed throughout adult stages and that STK17B is a target of PKC phosphorylation in the cerebellum. STK17B overexpression potentiates the morphological changes of Purkinje cells seen after PKC activation, suggesting that it is a downstream effector of PKC. A phosphorylation mimetic STK17B variant induced a marked reduction of Purkinje cell dendritic tree size, whereas the inhibition of STK17B with the novel compound 16 (Cpd16) could partially rescue the morphological changes of the Purkinje cell dendritic tree after PKC activation. These findings show that STK17B signalling is an important downstream effector of PKC activation in Purkinje cells. Furthermore, STK17B was identified as a molecule being transcriptionally downregulated in mouse models of SCA1, SCA7, SCA14 and SCA41. The reduced expression of STK17B in these mouse models might protect Purkinje cell dendrites from the negative effects of overactivated PKC signalling. Our findings provide new insights in the role of STK17B for Purkinje cell dendritic development and the pathology of SCAs.

## Graphical abstract

In a mouse model of SCA14 with constitutive activation of PKCγ signalling, we show that serine/threonine kinase 17b (STK17B) is strongly phosphorylated and is involved in the increased downstream signalling of PKCγ. Furthermore, STK17B was identified as a molecule being dysregulated in three more mouse models of diverse types of spinocerebellar ataxias (SCAs).

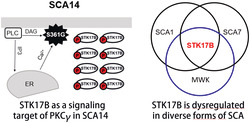

**Abbreviations**
Cpd16: compound 16
DAG: diacylglycerol
DRAK2: DAP kinase‐related apoptotic kinase 2
IP3: inositol 1,4,5‐trisphosphate
*Mwk*
: moonwalker
PKC: protein kinase C
PKD: protein kinase D
PLC: phospholipase C
PMA: phorbol myristate acetate
RGS8: regulator of G‐protein signalling 8
SCA: spinocerebellar ataxia
STK17B: serine/threonine kinase 17b
TRPC3: transient receptor potential cation channel subfamily c member 3

## 1 INTRODUCTION

The serine/threonine kinase STK17B, also known as DAP kinase‐related apoptosis‐inducing kinase 2 (DRAK2), was shown to be an important immunomodulator in T‐cell biology and is known to be a substrate of PKC isoforms in different cell types of the immune system (Friedrich et al., 2005; Kuwahara et al., 2008; Newton et al., 2011). PKC activity plays an important role in T‐cell activation and thymocyte selection. The PKC activator PMA strongly induced increased expression of STK17B, and inhibition of the PKC pathway can reduce the PMA‐induced enhanced expression of STK17B in CD4/CD8 double‐positive thymocytes, indicating an important role of PMA‐induced STK17B signalling in thymocytes (Friedrich et al., 2005). In our laboratory, we are interested in the role of protein kinase C gamma (PKCγ) for Purkinje cell dendritic development and for the pathogenesis of spinocerebellar ataxias (SCAs) (Shimobayashi & Kapfhammer, 2018). Activation of PKCγ results in a strong reduction of the Purkinje cell dendritic tree (Gugger et al., 2012), and a constitutive activation of PKCγ may be an important determinant of SCAs (Ji et al., 2014; Shimobayashi & Kapfhammer, 2021). At the same time, it is still not very well understood which molecules downstream of PKCγ activation are involved in the reduction of dendritic outgrowth in Purkinje cells. As STK17B is a well‐known target of PKC phosphorylation controlling cell differentiation in immune cells, we set out to study whether it might also be a target of PKCγ in cerebellar Purkinje cells and whether it might be involved in mediating the negative effects of PKCγ for Purkinje cell dendritic outgrowth. We show that STK17B is expressed in Purkinje cells and that it is at least in part mediating the effects of PKCγ for dendritic outgrowth. The transgenic expression of a phosphorylation mimetic STK17B variant in Purkinje cells resulted in impaired dendritic growth and a novel STK17B inhibitor compound (Cpd16) was able to inhibit STK17B activity and could partially protect Purkinje cell dendrites from retraction induced by PKC activation. We also found evidence that STK17B is dysregulated in diverse forms of SCA. Our findings identify STK17B signalling as a mediator of protein kinase C (PKC) activity in Purkinje cells, which might also be involved in the pathogenesis of SCAs.

## 2 MATERIALS AND METHODS

### 2.1 Animals

FVB mice were used for primary cerebellar cell cultures. The transgenic PKCγ(S361G) mice with FVB background used in this study were described earlier (Ji et al., 2014; Shimobayashi & Kapfhammer, 2017; Wu & Kapfhammer, 2021a). After 2 months, mice are considered adults, and the adult mice used in this study were 11 weeks old. All experiments were carried out in accordance with the EU Directive 2010/63/EU for the care and use of laboratory animals and were approved by the Veterinary Office of the Canton of Basel and permitted by Swiss authorities.

### 2.2 Immunostainings

Immunohistochemistry was performed as described before (Shimobayashi & Kapfhammer, 2017; Wu & Kapfhammer, 2021b, 2021c). Mice were sacrificed for cerebellar sections and perfused with 4% paraformaldehyde. Sagittal sections were cut with a cryostat (Leica) at 20 μm. Primary dissociated cerebellar cell cultures were fixed in 4% paraformaldehyde for 30 min at room temperature. All reagents were diluted in 100 mM phosphate buffer (PB). The sections were incubated with primary antibodies diluted in blocking solution (PB + 3% non‐immune goat serum + 0.3% Triton X‐100) overnight at 4°C, and dissociated cerebellar cultures were incubated for 1 h at room temperature. After washing with PB, the corresponding fluorescence conjugated secondary antibodies were added to the culture dishes in PB with 0.1% Triton X‐100 for 2 h at room temperature. The following primary antibodies were used: rabbit anti‐calbindin D‐28 K (1:500, Swant, Marly, Switzerland); rabbit anti‐DRAK2 (STK17B) (1:4000, Abcam); mouse anti‐calbindin D‐28 K (1:500, Swant, Marly, Switzerland). The following secondary antibodies were used: goat anti‐mouse Alexa 488 (1:500, Molecular Probes, Invitrogen); goat anti‐rabbit Alexa 488 (1:500, Molecular Probes, Invitrogen). Stained slices or sections were mounted with Mowiol (Sigma‐Aldrich, Buchs, Switzerland). Images were acquired with an Olympus AX70 microscope equipped with a SPOT Insight digital camera.

### 2.3 Cerebellar cell culture

Primary cerebellar cell cultures were prepared from neonatal mice as described before (Shimobayashi & Kapfhammer, 2017; Wu & Kapfhammer, 2021a, 2021d). Cerebellar cells were dissociated from postnatal Day 0 mice and plated on poly‐D‐lysine coated glass chambers. Indicated vectors were introduced into Purkinje cells by transfection with a Neon transfection system (Thermo Fisher) using the following settings: pulse voltage 1200 V, pulse width 30 ms, pulse rate 1. The cells were incubated in incubation medium (90% Dulbecco's modified Eagle medium/F‐12 nutrient medium, 1% N2 supplement, 1% Glutamax and 10% FBS). Two to four hours after transfection, 0.5 ml supplement containing 1% N2 and 1% Glutamax was added to each well. Half of the medium was then refreshed every 4 days. The media and supplements were supplied by Life Technologies (Zug, Switzerland). The cells were kept in culture for 2 weeks before fixation.

### 2.4 Pharmacological compounds

The following pharmacological compounds were added to the medium of Purkinje cells after 7 days: PMA (TOCRIS, Bio‐Techne AG). Cpd16, a novel inhibitor of STK17B, was synthetized according to the published data (Jung et al., 2016). The purification and quality control of the Cpd16 was performed by using high‐performance liquid chromatography (HPLC), liquid chromatography–mass spectrometry (LC‐MS) and nuclear magnetic resonance (NMR) spectroscopy. Cpd16 was produced, purified and supplied by WuXi AppTec (Wuhan, China).

### 2.5 Western blot

The samples were separated by SDS‐PAGE and blotted onto a nitrocellulose membrane. After blotting, the membranes were incubated with 5% BSA in TBS for 1 h and incubated with the specific primary antibodies. After washing with TBS‐T, the membranes were incubated with HRP‐labelled secondary antibodies. The proteins were visualized with ECL (Pierce, Thermo Scientific, Reinach, Switzerland). Alternatively, the membranes were incubated with IRDye secondary antibodies for 1 h. The proteins were quantified using an Odyssey system (LI‐COR Biosciences, Bad Homburg, Germany). HEK293T cells were transfected with the plasmid pCMV‐STK17B using Lipofectamine 3000 (Invitrogen) according to manufacturer's instructions and incubated for 24–48 h prior to harvest. The following primary antibodies were used for Western blots: rabbit anti‐DRAK2(STK17B) (1:1000, Abcam), mouse anti‐DRAK2(2C3) (1:2000, NOVUS), mouse anti‐actin (1:2000, Millipore), rabbit anti‐phospho‐(Ser) PKC substrate (1:1000, Cell Signaling), mouse anti‐GAPDH (1:4000, Proteintech). The following secondary antibodies were used in this study: Anti‐Mouse HRP conjugate antibody (1:10000, Promega); anti‐Rabbit HRP conjugate antibody (1:10000, Promega); IRDye 680LT Goat anti‐Rabbit IgG secondary antibody (1:10000, LICOR); IRDye 800CW Goat anti‐Mouse IgG secondary antibody (1:10000, LICOR). Spectra Multicolor Broad Range Protein Ladder (Thermo Scientific) was used in this study.

### 2.6 Transcriptome analysis

Data of genes whose expression is significantly altered in SCA1 and SCA7 mouse models were used from published data (Gatchel et al., 2008). Microarray data Purkinje cells of the moonwalker (*Mwk*) mouse model were used from a published study (Dulneva et al., 2015).

### 2.7 Quantitative analysis

The quantification of the size of Purkinje cell dendritic arbors was performed as described before (Shimobayashi & Kapfhammer, 2017; Wu & Kapfhammer, 2020, 2021a). The average value of the control Purkinje cells was normalized to 1. An image analysis program (ImageJ) was used to follow the contours of the Purkinje cell dendritic trees, which give the area of the dendritic tree. The mean fluorescence intensity of STK17B in the Purkinje cell nucleus and cytoplasm or dendrites was calculated, and the raw images were used for fluorescence intensity analysis. The data were analysed with GraphPad Prism software (San Diego, USA). The statistical significance of the differences in the parameters was assessed using the two‐tailed *t*‐test, the non‐parametric two‐tailed Mann–Whitney test or the ANOVA with Kruskal–Wallis test. The confidence intervals were 95% and statistical significance was assumed to be *P* < 0.05.

## 3 RESULTS

### 3.1 The PKCγ target STK17B is expressed in Purkinje cells

**FIGURE 1:**
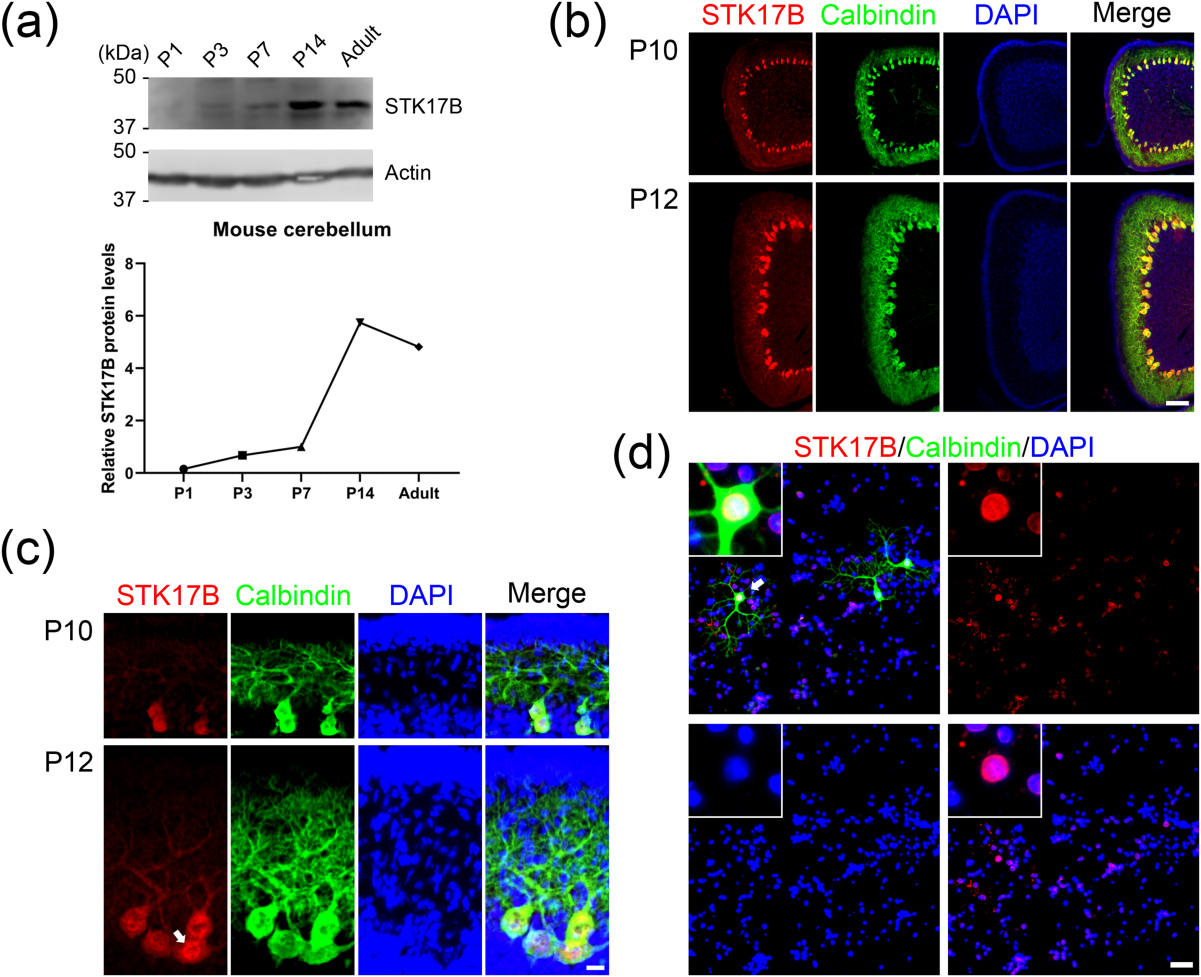
STK17B protein is expressed in Purkinje cells. (a) Western blots from mouse cerebellum at different postnatal stages. Top panel: STK17B expression is first detected at P3. Actin was used as loading control. Bottom panel: Quantification of protein levels from Western blots. Data are expressed as mean ± SD, with three independent biological samples for P1 (three or four pups for one sample), P3 and P7 (three or four pups for one sample) and two independent biological samples for P14 and adult. The mean value of STK17B protein level at P7 was set as 1, and values for the other stages were expressed relatives of this value. The mean values of P1, P3, P14 and adult were 0.1570 ± 0.1276, 0.6769 ± 0.3392, 5.745 ± 1.875 and 4.817 ± 1.722. (b) STK17B immunoreactivity (red signal) is present in cerebellar Purkinje cells (identified by anti‐calbindin, green) at P10 and P12. Scale bar is 100 μm. (c) Viewed at higher magnification, STK17B is mainly present in the soma of Purkinje cells at P10 and P12. Arrow indicates strong nuclear expression. Scale bar is 20 μm

STK17B is a well‐known target of various PKC isoforms in the immune system, but little is known about its presence and possible functions in the nervous system, in particular in cerebellar Purkinje cells that have a very strong expression of PKCγ. In order to better characterize the possible function of STK17B in the cerebellum, we studied the developmental expression profile of STK17B in the cerebellum of the mouse from postnatal Day 1 (P1) to adult by Western blot. There was no protein detectable at P1, and only weak signals were present at P3 and P7, with much stronger expression from P14 to adulthood (Figure 1a). In order to study the cellular expression of STK17B, sagittal cerebellar sections were analysed by immunohistochemistry at P10 and P12. STK17B protein was most strongly expressed in Purkinje cells, and only little expression was seen in other cerebellar cells (Figure 1b). At higher magnification, STK17B was present in the cell body and dendrites of Purkinje cells. We also found a strong nuclear expression of STK17B in Purkinje cells by qualitative observation (Figure 1c). Primary dissociated cerebellar cultures confirmed that STK17B immunoreactivity was present in Purkinje cells, but now also other cerebellar cells were positive. In Purkinje cells, the expression was almost exclusively restricted to the nucleus (Figure 1d).

### 3.2 PKC substrate STK17B phosphorylation is increased in Purkinje cells from a mutant mouse (PKCγ‐S361G) with constitutive PKC activation

**FIGURE 2:**
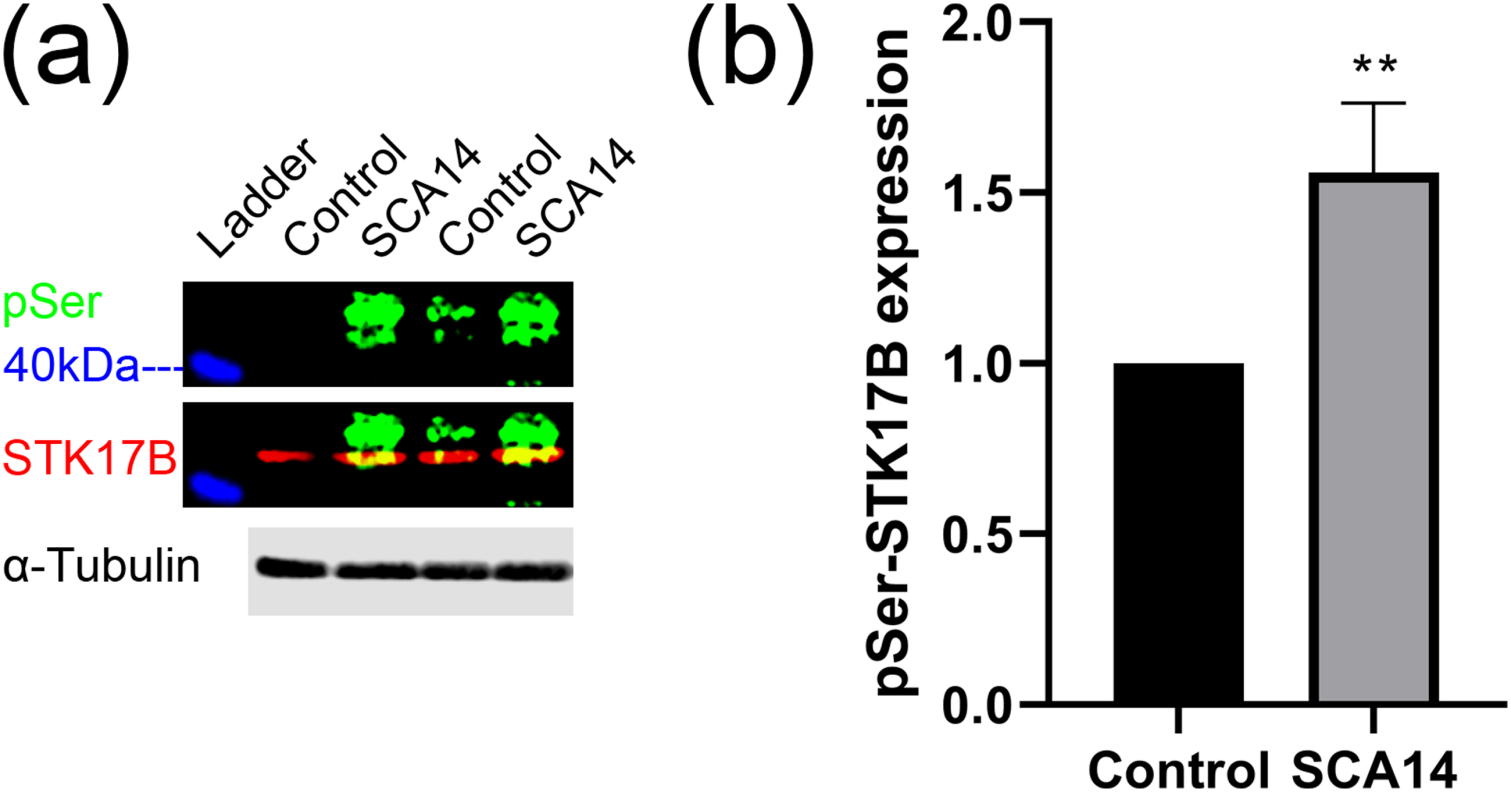
Constitutive PKCγ‐S361G mutant induces increased phosphorylation of STK17B. (a) Identification of STK17B protein with a size just above the 40‐kDa label of the protein ladder. (b) p‐Ser phosphorylation of STK17B is increased in the Purkinje cells of a SCA14 mouse model with constitutive PKCγ activity (PKCγ‐S361G)

STK17B has previously been identified as a downstream signalling molecule of PKC isoforms. PMA‐induced PKC activation was shown to induce STK17B phosphorylation in immune cells (Friedrich et al., 2005; Kuwahara et al., 2008; Newton et al., 2011). Importantly, PKCγ specifically phosphorylates Ser350 of rat STK17B, which corresponds to Ser351 of mouse STK17B (Kuwahara et al., 2008). Because no antibody specific for phosphorylated STK17B is available, we used a fluorescent Western blot detection strategy (including 600, 700 and 800 nm) and were able to detect two protein targets and a 40‐kDa protein ladder simultaneously through different channels. We detected phospho‐Ser using a commercial rabbit antibody against p‐Ser PKC substrates. This antibody was produced by immunizing with phospho‐PKC substrate peptides and was able to recognize endogenous PKC substrate only when phosphorylated at serine residues. A mouse STK17B antibody was chosen to detect the STK17B protein. Spectra multicolour broad range protein ladder was used, and the 40‐kDa protein ladder could be detected by 600‐nm channel. Using this approach, we assayed STK17B phosphorylation. At the position above 40 kDa (blue) of the protein ladder, the bands of STK17B (red) and p‐Ser phosphorylation (green) were detected on the Western blotting membrane and merged. We found that p‐Ser phosphorylation was increased (control vs. SCA14: 1.000 vs. 1.558 ± 0.2043, three independent experiments, *t* = 4.736, *df* = 4, *P* = 0.0091, *t*‐test) in a mouse model with constitutive PKCγ activity (PKCγ‐S361G) in Purkinje cells (Ji et al., 2014), indicating that in cerebellar Purkinje cells, STK17B is indeed a phosphorylation target of PKCγ (Figure 2a,b).

### 3.3 STK17B phosphorylation impairs dendritic growth of Purkinje cells

**FIGURE 3:**
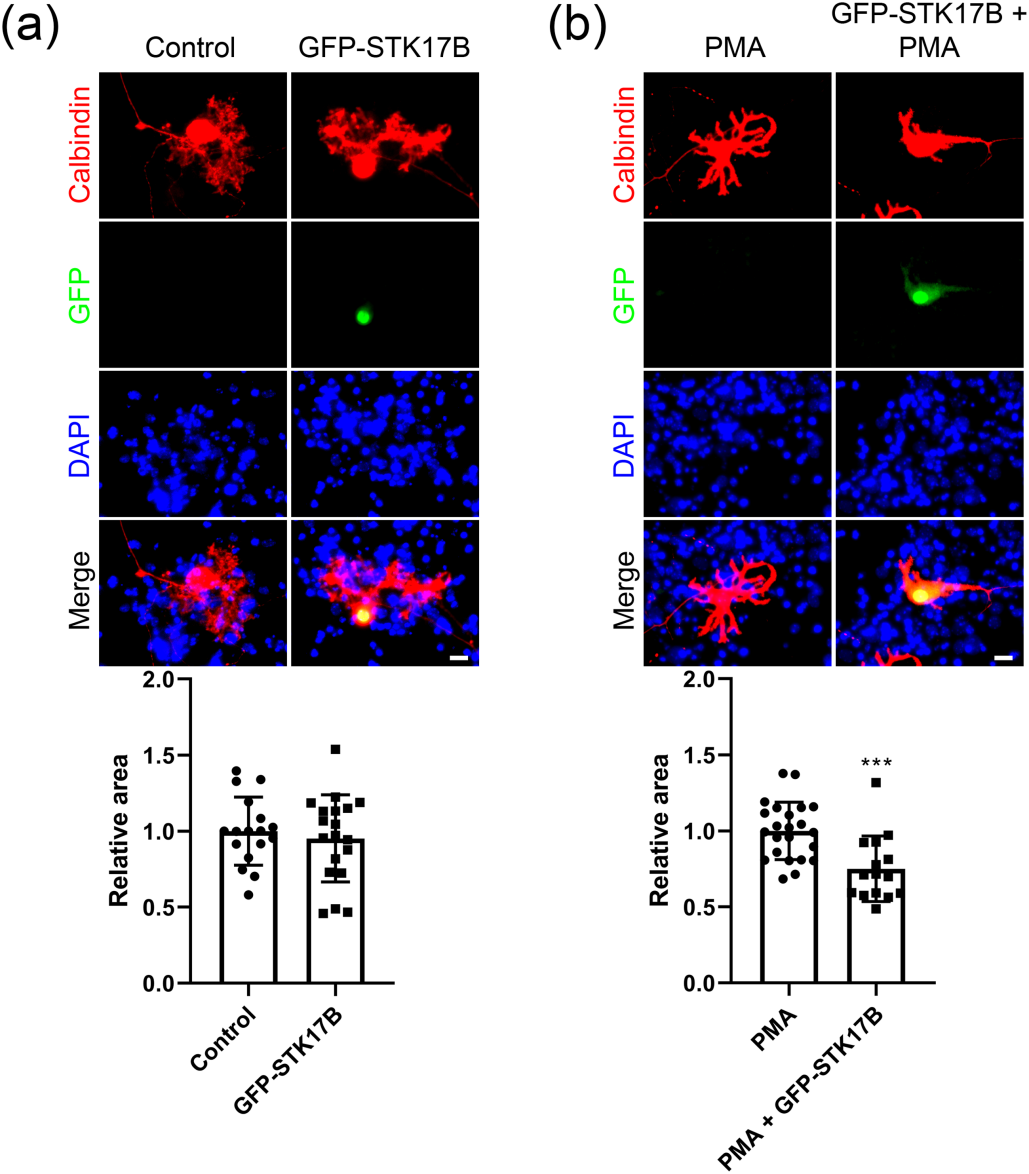
Overexpression of STK17B potentiates the PMA effect on Purkinje cell dendritic morphology. (a) Purkinje cells were identified by anti‐calbindin staining (red), and cell nuclei were stained by DAPI (blue). Overexpressed STK17B in Purkinje cells in dissociated cerebellar cultures had little effect on Purkinje cell dendritic morphology. (b) STK17B transfection potentiates the effect of treatment with the PKC activator PMA on the Purkinje cell dendritic tree. Representative images of control and STK17B‐transfected Purkinje cells under 0.75–1.25 nM PMA treatment. After STK17B transfection, dendritic expansion is reduced as compared with PMA treatment alone. Data are expressed as mean ± SD. Scale bar is 20 μm

Increased activity of PKCγ in cerebellar Purkinje cells is associated with reduced dendritic differentiation and growth, and Purkinje cells in organotypic slice cultures either with activation of PKCγ by the phorbol ester PMA or in slice cultures from PKCγ‐S361G mice only develop stunted dendritic trees (Ji et al., 2014). When we overexpressed STK17B in Purkinje cells in dissociated cerebellar cultures, it had little effect on Purkinje cell dendritic morphology, indicating that wild‐type STK17B has no negative effect on Purkinje cell dendritic development (1.000 ± 0.2237 vs. 0.9524 ± 0.2862; *n* = 17, 19; *P* = 0.7482, two‐tailed Mann–Whitney *U* test) (Figure 3a). However, when PKC was activated by treatment with low concentrations of PMA, overexpression of STK17B enhanced the dendritic phenotype (1.000 ± 0.1884 vs. 0.7505 ± 0.2156, *n* = 23, 15; *P* = 0.0004, two‐tailed Mann–Whitney *U* test) (Figure 3b), suggesting that it may be involved in downstream signalling from PKCγ and that phosphorylated STK17B may have a negative effect on Purkinje cell dendritic growth.

### 3.4 A phospho‐mimetic form of STK17B interferes with Purkinje cell dendritic development

**FIGURE 4:**
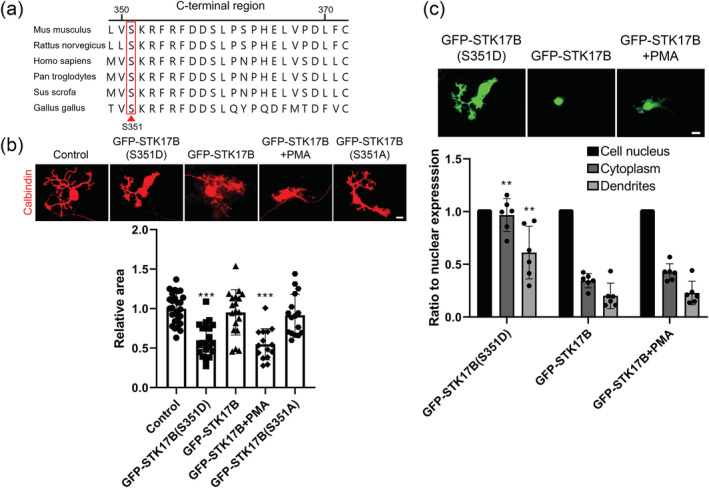
Increased phosphorylation of STK17B inhibits Purkinje cell dendritic development. (a) Conservation of S351 (in red) in the C‐terminal region of STK17B among species. The amino acid sequence is from accession numbers *Mus musculus* NP_598571.2, *Rattus norvegicus* NP_596883.1, *Homo sapiens* NP_004217.1, *Pan troglodytes* XP_001168212.1, *Sus scrofa* XP_020930482.1 and *Gallus gallus* NP_001026420.1. (b) Representative images of Purkinje cells after control or STK17B(S351D) or wild‐type STK17B or wild‐type STK17B plus low concentration PMA or STK17B(S351A) transfection. Transfection of phospho‐mimetic STK17B(S351D) or wild‐type STK17B plus PMA but not wild‐type STK17B or STK17B(S351A) inhibits Purkinje cell dendritic development. (c) GFP‐STK17B(S351D) is largely distributed in the cytoplasm and dendrites of Purkinje cells, whereas wild type GFP‐STK17B mainly expressed in the cell nucleus. The ratio of GFP‐fused STK17B(S351D) or wild‐type STK17B expression of the indicated regions to the nucleus in cells was calculated. Expression of GFP‐fused STK17B(S351D) or wild‐type STK17B in nucleus was normalized as 1

Phosphorylation of serine350 of rat STK17B by PKC in the C‐terminal region has been reported to affect STK17B nuclear localization in cell line experiments (Kuwahara et al., 2008). By comparison of the C‐terminal sequence across various species, we found a conserved C‐terminal region in STK17B protein corresponding to serine351 (Ser351) of mouse STK17B (Figure 4a). In order to test whether activation of STK17B signalling does affect Purkinje cell dendritic development, we constructed plasmids of wild‐type STK17B, a phospho‐mimetic S351D form of mouse STK17B and a non‐phosphorylatable form S351A for transfection in Purkinje cells. We found that transfection of neither the wild‐type form nor of the non‐phosphorylatable form STK17B‐S351A had a significant effect of Purkinje cell dendritic development. In contrast, transfection of the phospho‐mimetic S351D form of STK17B strongly interfered with Purkinje cell dendritic development (1.000 ± 0.1908, 0.6017 ± 0.2059, 0.9524 ± 0.2862, 0.9174 ± 0.2623; *n* = 27, 20, 19, 16; *P* < 0.001, Kruskal–Wallis test) (Figure 4b). Interestingly, the intracellular distribution of the wild‐type STK17B form showed a predominantly nuclear localization, whereas STK17B(S351D) was widely distributed also in the cytoplasm and dendrites (Figure 4c). We compared the relative intensity of STK17B expression in the nucleus versus cytoplasm and dendrites. The measurements confirmed the predominant localization of wild‐type STK17B to the nucleus, whereas GFP‐STK17B(S351D) was widely distributed in the cytoplasm and in the dendrites of Purkinje cells (cytoplasm: 0.9673 ± 0.1556 vs. 0.3454 ± 0.06840; dendrites: 0.6117 ± 0.2504 vs. 0.2007 ± 0.1210; *n* = 6; *P* < 0.01, two‐tailed Mann–Whitney *U* test) (Figure 4c). In Purkinje cells treated with the PKC agonist PMA, we also found more cytoplasmic GFP‐STK17B, but this was not as pronounced as with STK17B‐S351D and did not reach statistical significance (*P* = 0.3939 in two‐tailed Mann–Whitney *U* test) (Figure 4c). Our results indicate that enhanced STK17B phosphorylation and activity in Purkinje cells are impeding dendritic development and are promoting a more cytoplasmic distribution.

### 3.5 Inhibiting STK17B activity by Cpd16 partially rescues Purkinje cell dendritic growth

**FIGURE 5:**
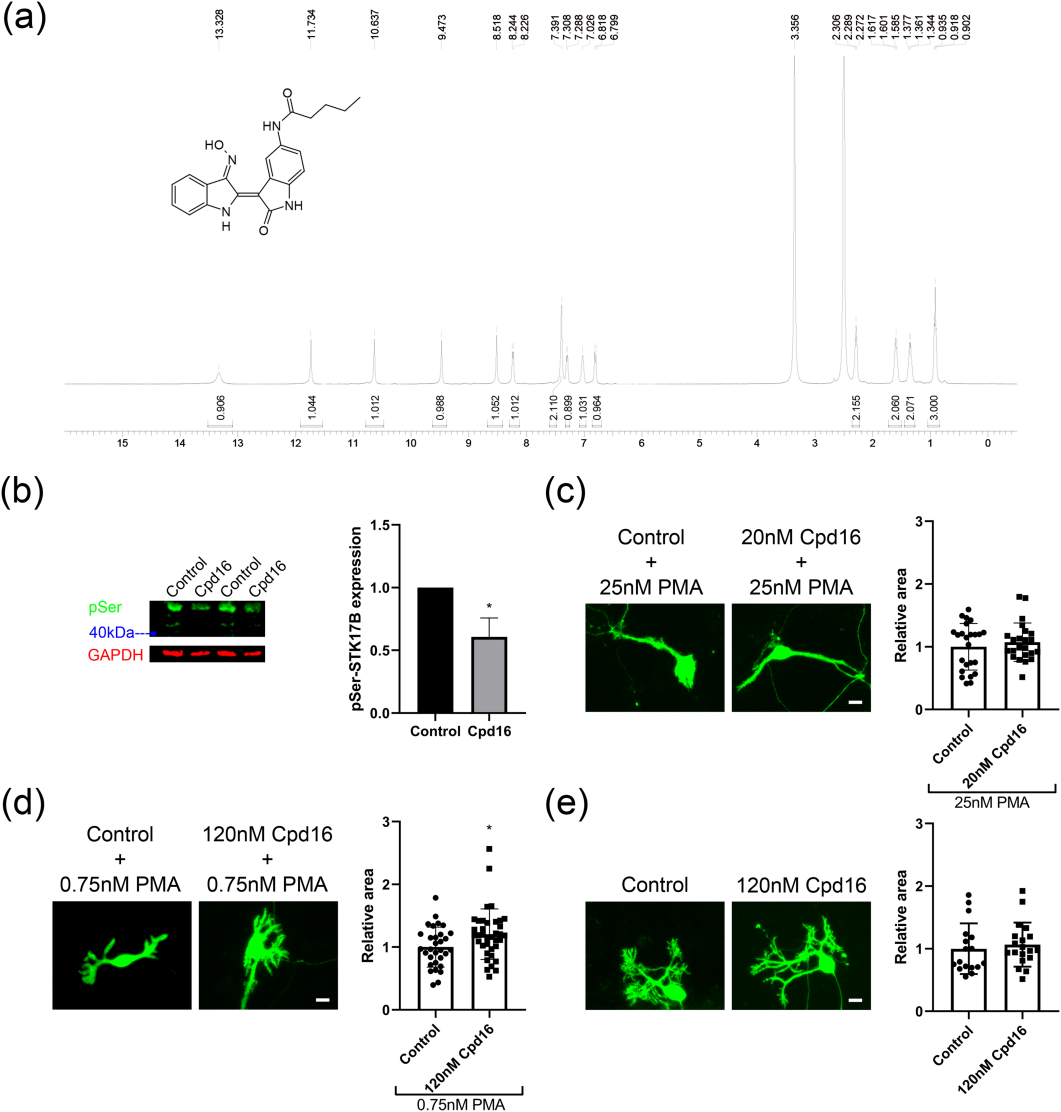
STK17B inhibitor protects the Purkinje cell dendritic tree from activation of PKC signalling. (a) The structural information of synthesized compound 16 (Cpd16) was confirmed by a nuclear magnetic resonance (NMR) spectroscopy‐based approach. (b) p‐Ser phosphorylation of STK17B is decreased in the HEK293T cells treated with 16 μM Cpd16 for 24 h. (c) No effect of Cpd16 treatment on Purkinje cell dendritic expansion after treatment with 25 nM PMA. The control + 25 nM PMA was set as 1. (d) Cpd16 treatment partially protects from PMA of low concentration induced reduction of dendritic expansion. The mean values of the Purkinje cell dendritic area were normalized to the control. (e) Cpd16 treatment did not significantly affect dendritic growth of Purkinje cells. Data are expressed as mean ± SD. Scale bars are 20 μm. Purkinje cells were stained with anti‐calbindin (green)

Recently, some indirubin derivatives have been reported to be specific inhibitors of STK17B (Jung et al., 2016). The compound 16 (Cpd16) has been suggested as being the most potent inhibitor of STK17B. In order to assess the Cpd16 effect in physiological experiments, Cpd16 was synthesized, and the structure of Cpd16 was determined by the NMR spectroscopy (Figure 5a). HPLC and LC‐MS confirmed the correct structure of synthesized Cpd16 (Figures S2–S7). In HEK293T cells transfected with STK17B, phosphorylated STK17B was detected in Western blots with a size of just above the 40‐kDa label (Figure 5b). We transfected HEK293T cells with STK17B and examined STK17B phosphorylation levels in the cells treated with Cpd16. We found that inhibition of STK17B with Cpd16 could reduce STK17B phosphorylation in HEK293T cells (1.000 vs. 0.6072 ± 0.1513; three independent experiments; *t* = 4.498, *P* = 0.0108, two‐tailed *t*‐test.) (Figure 5b). We wondered if inhibiting STK17B activity could modify the effect of PMA‐induced PKC activation. When Purkinje cells were treated with 25 nM PMA, 20 nM Cpd16 could not rescue Purkinje cell dendritic morphology (1.000 ± 0.3722 vs. 1.074 ± 0.3064; n = 24, 23; *P* = 0.7598; two‐tailed Mann–Whitney *U* test) (Figure 5c). We then reduced the PMA concentration to 0.75 nM PMA and increased the Cpd16 concentration to 120 nM Cpd16. Under these conditions, we were able to find a small improvement of Purkinje cell dendritic morphology, which reached statistical significance (1.000 ± 0.3176 vs. 1.206 ± 0.4025; *n* = 31, 37; *P* = 0.0252; two‐tailed Mann–Whitney *U* test) (Figure 5d). The treatment of Purkinje cells with 120 nM Cpd16 by itself did not obviously affect development of Purkinje cells (1.000 ± 0.4057 vs. 1.065 ± 0.3528; *n* = 17, 19; *P* = 0.4147; two‐tailed Mann–Whitney *U* test) (Figure 5e).

### 3.6 STK17B function might be involved in diverse forms of SCA

**FIGURE 6:**
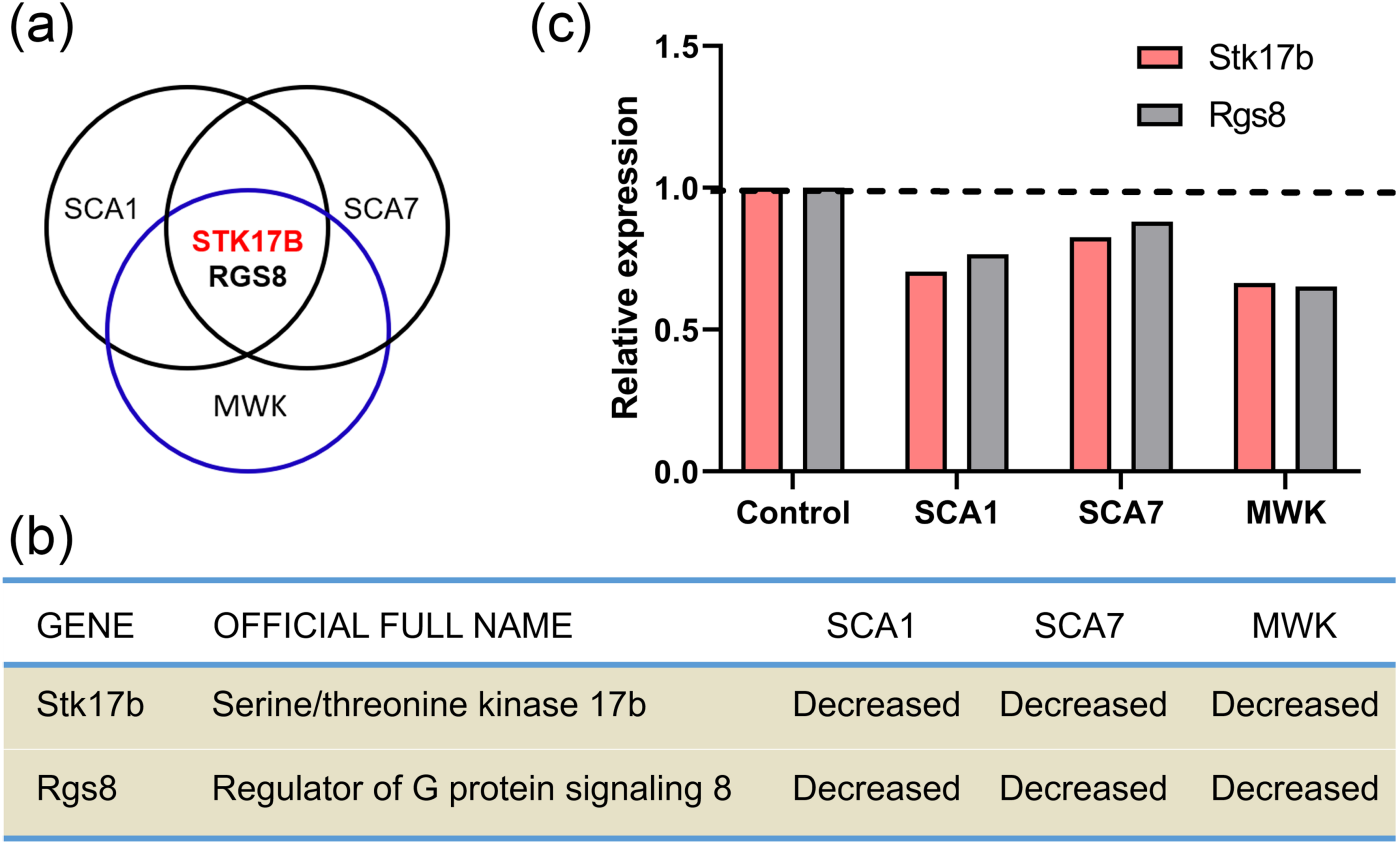
Comparison of transcriptional changes in SCA1, SCA7 and *Mwk* mice. (a) Venn diagram of transcriptional changes using published microarray data of SCA1, SCA7 and moonwalker (*Mwk*) mice. (b) Overlap of gene sets between SCA1, SCA7 and *Mwk* mice. (c) Validation of two overlapping genes STK17B and RGS8 shows reductions of transcript expression. All the data of SCA1, SCA7 and *Mwk* mice are based on published microarray data sets

STK17B was also identified as a protein being transcriptionally regulated in different forms of SCA. Global transcriptional analysis in the cerebellum of two SCA mouse models, SCA1 and SCA7 mice, at early symptomatic disease stage has identified 27 genes dysregulated in both mouse models (Gatchel et al., 2008). We now combined the results from this analysis with a more recent study in moonwalker (*Mwk*) mice. *Mwk* mouse is a spontaneous mutant mouse that shows abnormal Purkinje cell development and cerebellar ataxia due to increased calcium influx to Purkinje cells through mutated TRPC3 channels (Becker et al., 2009), and TRPC3 was also identified as the gene causing SCA41 (Fogel et al., 2015). Developing Purkinje cells from *Mwk* mice were isolated by laser‐capture microdissection to test transcriptomic changes in Purkinje cells of 18‐day‐old *Mwk* mice (Dulneva et al., 2015). When we compared 634 dysregulated genes in Purkinje cells of *Mwk* mice (Dulneva et al., 2015) with the 27 common genes from SCA1 and SCA7 mice (Gatchel et al., 2008), STK17B was one of only two mRNAs, STK17B and RGS8, which were found to be dysregulated in all three mouse models (Figure 6a–c). Our findings about RGS8 have been published previously (Wu & Kapfhammer, 2021a). We then assayed the expression of STK17B protein in our own mouse model of SCA14, the PKCγ‐S361G mice (Ji et al., 2014). STK17B immunoreactivity was significantly decreased to 0.83 ± 0.21‐fold lower expression in Purkinje cells from transgenic mice versus GFP‐negative Purkinje cells from control mice present in the same culture well (1.000 ± 0.2514 vs. 0.8303 ± 0.2117; *n* = 28, 21; *P* = 0.0124, Mann–Whitney *U* test) (Figure S1). These results show that STK17B protein is dysregulated in mouse models of diverse SCAs. In all these mouse models, a reduced expression of STK17B was found. These results indicate that STK17B might be involved in both Purkinje cell development and the pathology of SCAs.

## 4 DISCUSSION

In this study, for the first time, we explored the role of STK17B in neurons, in particular cerebellar Purkinje cells. STK17B is a serine/threonine kinase belonging to the death‐associated protein kinase family. Previous studies focused on the role of STK17B in the immune system due to its high expression in thymus (Friedrich et al., 2005; Gatzka et al., 2009; Kuwahara et al., 2008; Newton et al., 2011; Ramos et al., 2008). STK17B was shown to be a substrate of PKC isoforms in different immune cell types (Friedrich et al., 2005; Kuwahara et al., 2008; Newton et al., 2011). PKC activity plays an important role in T‐cell activation and thymocyte selection. The PKC activator PMA strongly induced increased expression of STK17B, and inhibition of the PKC pathway can reduce the PMA‐induced enhanced expression of STK17B in CD4/CD8 double‐positive thymocytes, indicating an important role of PMA‐induced STK17B signalling in thymocytes (Friedrich et al., 2005). In HEK293T cells, co‐expression of a dead PKCμ (also known as PKD) mutant and STK17B reduced the phosphorylation of STK17B, and PMA increased phosphorylation of STK17B, suggesting that PKC activity promotes STK17B function (Newton et al., 2011). However, little is known about STK17B functions in the nervous system. We studied PMA‐induced phosphorylation and activation of STK17B functions in Purkinje cells, and our findings agree with the previous studies that STK17B is a target of PKC and PKC activity increases STK17B function. It was shown earlier that PKCγ specifically phosphorylates Ser350 of rat STK17B, corresponding to Ser351 of mouse STK17B (Kuwahara et al., 2008). We have now shown that the expression of a phosphorylation mimetic STK17B mutant, but not of wild type of STK17B, induced a reduction of Purkinje cell dendritic growth. We show for the first time an important biological function of STK17B in the nervous system. As in Purkinje cells there is a very high expression of PKCγ, STK17B is likely to act as a downstream mediator of PKCγ activity.

Recently, indirubin derivatives have been identified as a novel class of STK17B inhibitors, and Cpd16 exhibited the most potent inhibitory activity against STK17B (Jung et al., 2016). We confirmed that the activation of STK17B was reduced with treatment of Cpd16 in HEK293T cells. We further found that Cpd16 could partly rescue the Purkinje cell morphology induced by a low concentration of PMA. These experiments confirm the role of STK17B as an important downstream mediator of PKC signalling during Purkinje cell dendritic development.

In another recent study, the phosphorylation of CRMP2 was identified as another downstream target of PKC and a potential downstream mediator of PKC activation in cerebellar Purkinje cells (Winkler et al., 2020). Interestingly, overexpression of either wild‐type STK17B or wild‐type CRMP2 transcripts in Purkinje cells in dissociated cultures did not affect Purkinje cell dendritic outgrowth, but transfection of the respective phospho‐mimetic variants had a strong effect on the development of the Purkinje cell dendritic tree underlining the importance of phosphorylation rather than just increased or decreased expression for the function of these molecules.

**FIGURE 7:**
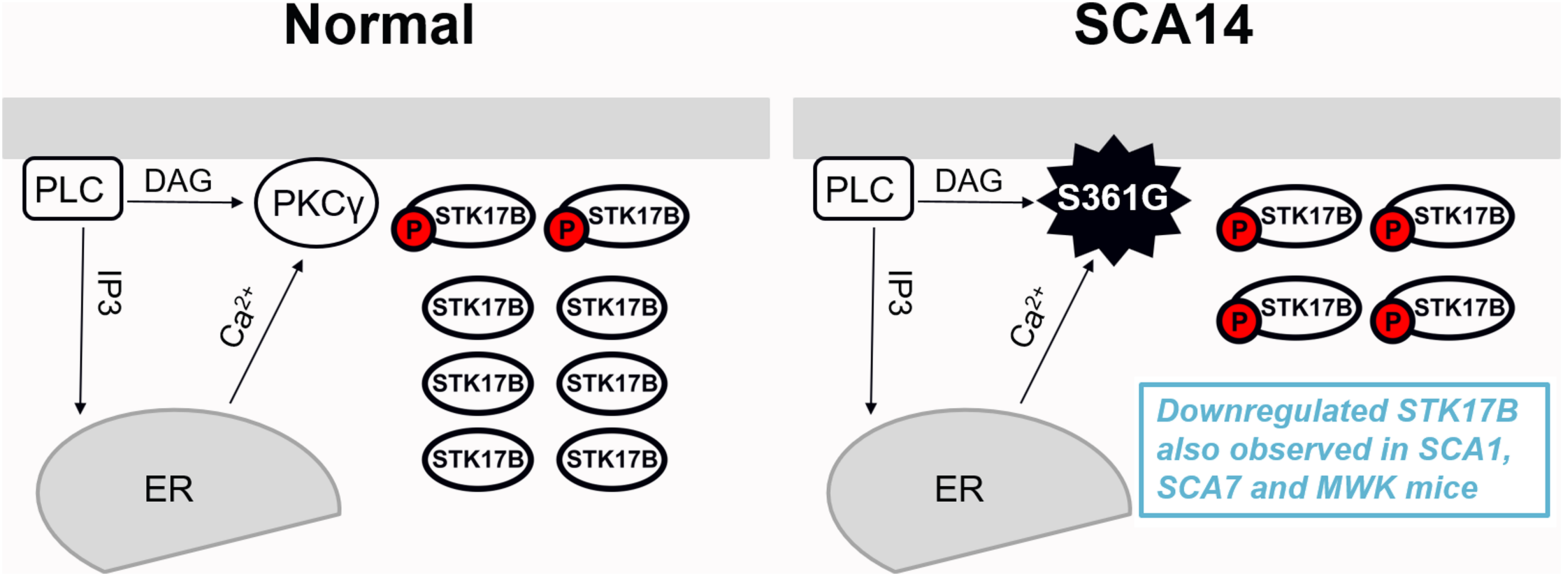
A possible model of the functional effect of STK17B. STK17B signalling in the normal situation (left panel). Phosphoinositide phospholipase C (PLC) is activated to produce inositol 1,4,5‐trisphosphate (IP3) and diacylglycerol (DAG). PKCγ is activated by the combination of DAG and Ca^2+^ to induce the phosphorylation of downstream substrates. In the SCA14 (right panel), PKCγ(S361G) is thought to be constitutively active mutant. Decreased expression of STK17B may be a protective reaction for the activation of STK17B by increased PKC activity. Downregulated STK17B has been reported in several SCAs implicating dysregulation of the STK17B signalling within the pathogenesis of SCAs

STK17B was also identified as a molecule possibly associated with SCA pathogenesis based on its transcriptional regulation in diverse mouse models of SCAs, specifically SCA1, SCA7 and SCA41 (Dulneva et al., 2015; Gatchel et al., 2008). We could confirm a reduced expression on the protein level in our own mouse model of SCA14 (Ji et al., 2014), further supporting a relationship of dysregulation of STK17B signalling and pathology of SCAs. In this mouse model, the mutated PKCγ(S361G) is thought to be a constitutively active mutant (Adachi et al., 2008), and we present evidence that STK17B is a target of PKCγ phosphorylation in Purkinje cells and a mediator of increased PKCγ activity with respect to Purkinje cell dendritic development. These findings in Purkinje cells are in agreement with a report showing that a constitutively active PKCμ (also known as PKD) mutant strongly causes STK17B activation and phosphorylation in cell line experiments (Newton et al., 2011). The reduced expression of STK17B in the PKCγ(S361G) mouse model could be explained by a compensatory reduction of STK17B expression in the presence of constitutive PKC activation. Such a reduction of STK17B expression in Purkinje cells could be viewed as a protective reaction counterbalancing the increased phosphorylation and activation of STK17B by increased PKC activity (Figure 7). In the moment, it is not known whether the reduction of STK17B expression in the other mouse models (SCA1, SCA7 and *MWK*/SCA41) is related to an increased PKCγ activity or whether it is due to different mechanisms. Additional studies will be needed to better define the role of STK17B in these mouse models and find explanations for its reduced expression.

## 5 CONCLUSION

STK17B is strongly expressed in Purkinje cells starting at early postnatal development, and it functions as a downstream mediator of PKC activity. Transfection of an STK17B variant mimicking the phosphorylated form impedes Purkinje cell dendritic development similar to increased PKC activity itself, and inhibition of STK17B by the novel inhibitor Cpd16 could partially protect Purkinje cell dendrites from increased PKC activity. These findings provide a first evaluation of the function of STK17B in the nervous system, in particular for Purkinje cell development and SCA pathology.

## CONFLICT OF INTEREST

The authors have no financial conflicts of interest.

## AUTHOR CONTRIBUTIONS

Q.‐W.W. conceived the research, conducted the experiments and data analyses and wrote the manuscript. J.P.K. supervised the study and revised the manuscript. Both authors were involved in discussions on the final manuscript. All authors read and approved the final manuscript.

### PEER REVIEW

The peer review history for this article is available at https://publons.com/publon/10.1111/ejn.15465.

## Supporting information


**FIGURE S1** Decreased STK17B expression in Purkinje cells of the SCA14 S361G mouse model. (a) All reported SCA14‐associated mutations in PKCγ are listed. In this study the transgenic mouse model of S361G in the kinase domain of PKCγ is used. (b) In the transgenic PKCγ(S361G) mouse line, Purkinje cell specific expression of the transgene is achieved by a bidirectional CMV promoter which expresses both GFP and mutant PKCγ. This expression is under the control of a Tetracycline response element (TRE) which will only will start transcription in the presence of the tet TransActivator (tTA), Tet‐off system. The tTA protein is expressed in a second transgenic locus under the control of the L7 promoter which only will be active in Purkinje cells. Only in mice which are double transgenic for GFP‐TRE‐PKCγ(S361G) and L7‐tTA, Purkinje cells will show expression of GFP and mutant PKCγ. (c) The transgenic Purkinje cells were identified by GFP staining in mixed dissociated cultures and STK17B protein expression was quantified on GFP‐positive Purkinje cells from transgenic mice versus GFP‐negative Purkinje cells from control mice present in the same culture well. The mean value of STK17B expression for SCA14 was decreased 0.8303 ± 0.2117 fold compared to control cells. The n was 28 for control and 21 for SCA14, and the difference in expression was significant with P = 0.0124 (*P < 0.05) in the two‐tailed Mann–Whitney test. Data are expressed as mean ± SD. Scale bar is 20 μm.
**Figure S2** A schematic diagram of the steps of synthesis used in this study.
**Figure S3** LC–MS was used to detect and characterize the Cpd16 and a summary of results.
**Figure S4** LC–MS report.
**Figure S5** HPLC report at 220 nm and a summary of results.
**Figure S6** HPLC report at 254 nm and a summary of results.
**Figure S7** HPLC report at 215 nm and a summary of results.

## Data Availability

The data that support the findings of this study are available from the corresponding author upon reasonable request.
