# Supplementary material for: Serine/threonine kinase 17b (STK17B) signalling regulates Purkinje cell dendritic development and is altered in multiple spinocerebellar ataxias
Source: Eur J Neurosci. 2021 Sep 29;54(7):6673–84. doi: 10.1111/ejn.15465 (PMC9292345; doi:10.1111/ejn.15465)
Supplement: Supplementary file 1 — FIGURE S1 Decreased STK17B expression in Purkinje cells of the SCA14 S361G mouse model. (a) All reported SCA14‐associated mutations in PKCγ are listed. In this study the transgenic mouse model of S361G in the kinase domain of PKCγ is used. (b) In the transgenic PKCγ(S361G) mouse line, Purkinje cell specific expression of the transgene is achieved by a bidirectional CMV promoter which expresses both GFP and mutant PKCγ. This expression is under the control of a Tetracycline response element (TRE) which will only will start transcription in the presence of the tet TransActivator (tTA), Tet‐off system. The tTA protein is expressed in a second transgenic locus under the control of the L7 promoter which only will be active in Purkinje cells. Only in mice which are double transgenic for GFP‐TRE‐PKCγ(S361G) and L7‐tTA, Purkinje cells will show expression of GFP and mutant PKCγ. (c) The transgenic Purkinje cells were identified by GFP staining in mixed dissociated cultures and STK17B protein expression was quantified on GFP‐positive Purkinje cells from transgenic mice versus GFP‐negative Purkinje cells from control mice present in the same culture well. The mean value of STK17B expression for SCA14 was decreased 0.8303 ± 0.2117 fold compared to control cells. The n was 28 for control and 21 for SCA14, and the difference in expression was significant with P = 0.0124 (*P < 0.05) in the two‐tailed Mann–Whitney test. Data are expressed as mean ± SD. Scale bar is 20 μm. Figure S2 A schematic diagram of the steps of synthesis used in this study. Figure S3 LC–MS was used to detect and characterize the Cpd16 and a summary of results. Figure S4 LC–MS report. Figure S5 HPLC report at 220 nm and a summary of results. Figure S6 HPLC report at 254 nm and a summary of results. Figure S7 HPLC report at 215 nm and a summary of results. [file EJN-54-6673-s001.pdf]

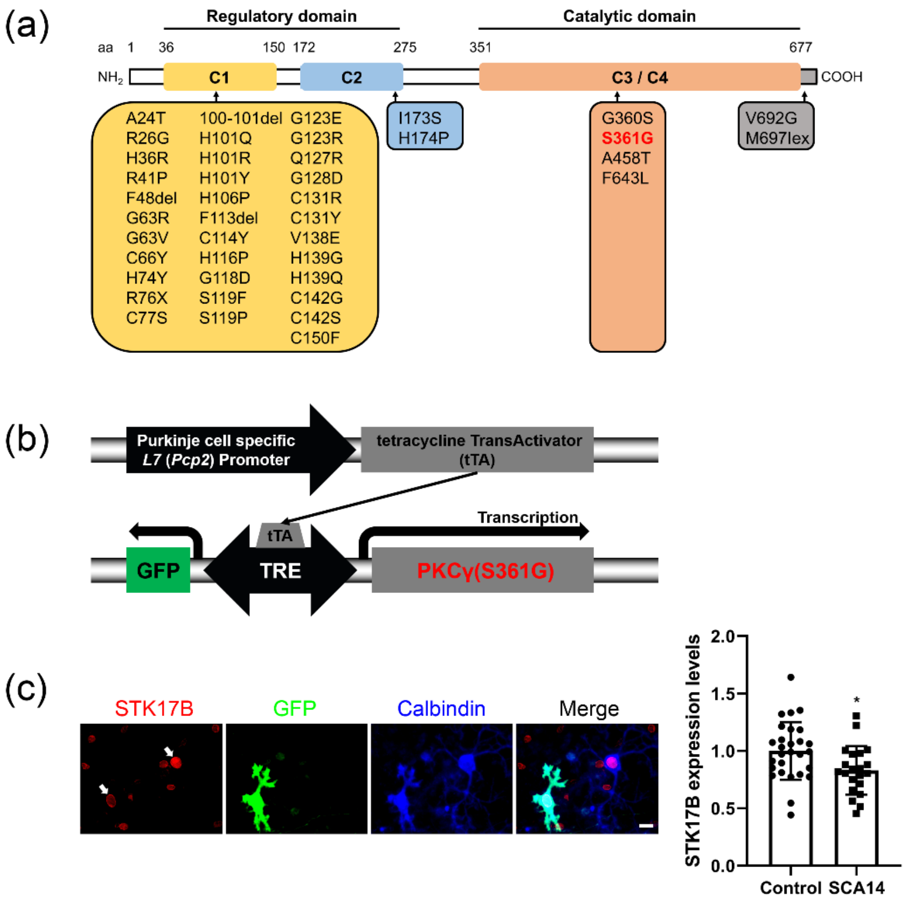

FIGURE S1 Decreased STK17B expression in Purkinje cells of the SCA14 S361G mouse model. (a) All reported SCA14-associated mutations in PKCγ are listed. In this study the transgenic mouse model of S361G in the kinase domain of PKCγ is used. (b) In the transgenic PKCγ(S361G) mouse line, Purkinje cell specific expression of the transgene is achieved by a bidirectional CMV promoter which expresses both GFP and mutant PKCγ. This expression is under the control of a Tetracycline response element (TRE) which will only will start transcription in the presence of the tet TransActivator (tTA), Tet-off system. The tTA protein is expressed in a second transgenic locus under the control of the L7 promoter which only will be active in Purkinje cells. Only in mice which are double transgenic for GFP-TRE-PKCγ(S361G) and L7-tTA, Purkinje cells will show expression of GFP and mutant PKCγ. (c) The transgenic Purkinje cells were identified by GFP staining in mixed dissociated cultures and STK17B protein expression was quantified on GFP-positive Purkinje cells from transgenic mice versus GFP-negative Purkinje cells from control mice present in the same culture well. The mean value of STK17B expression for SCA14 was decreased 0.8303 ± 0.2117 fold compared to control cells. The n was 28 for control and 21 for SCA14, and the difference in expression was significant with P = 0.0124 (*P < 0.05) in the two-tailed Mann-Whitney test. Data are expressed as mean ± SD. Scale bar is 20 μm.

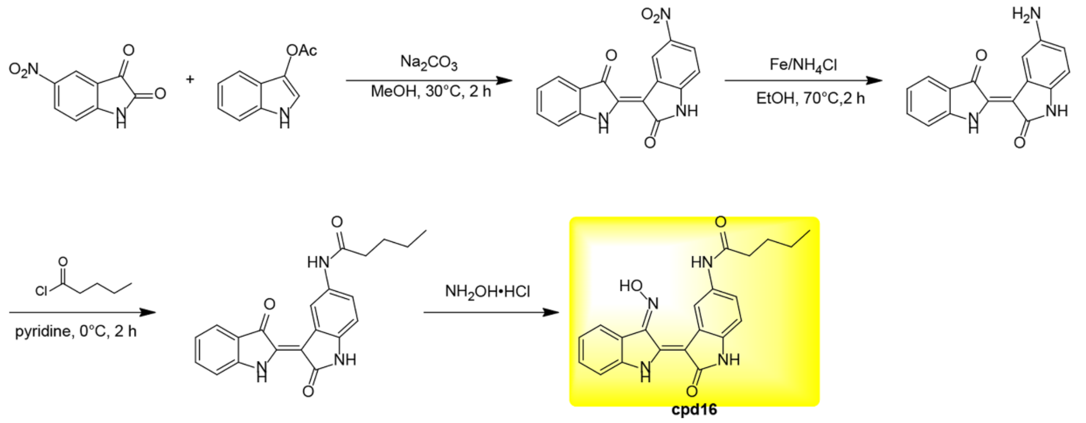

FIGURE S2 A schematic diagram of the steps of synthesis used in this study.

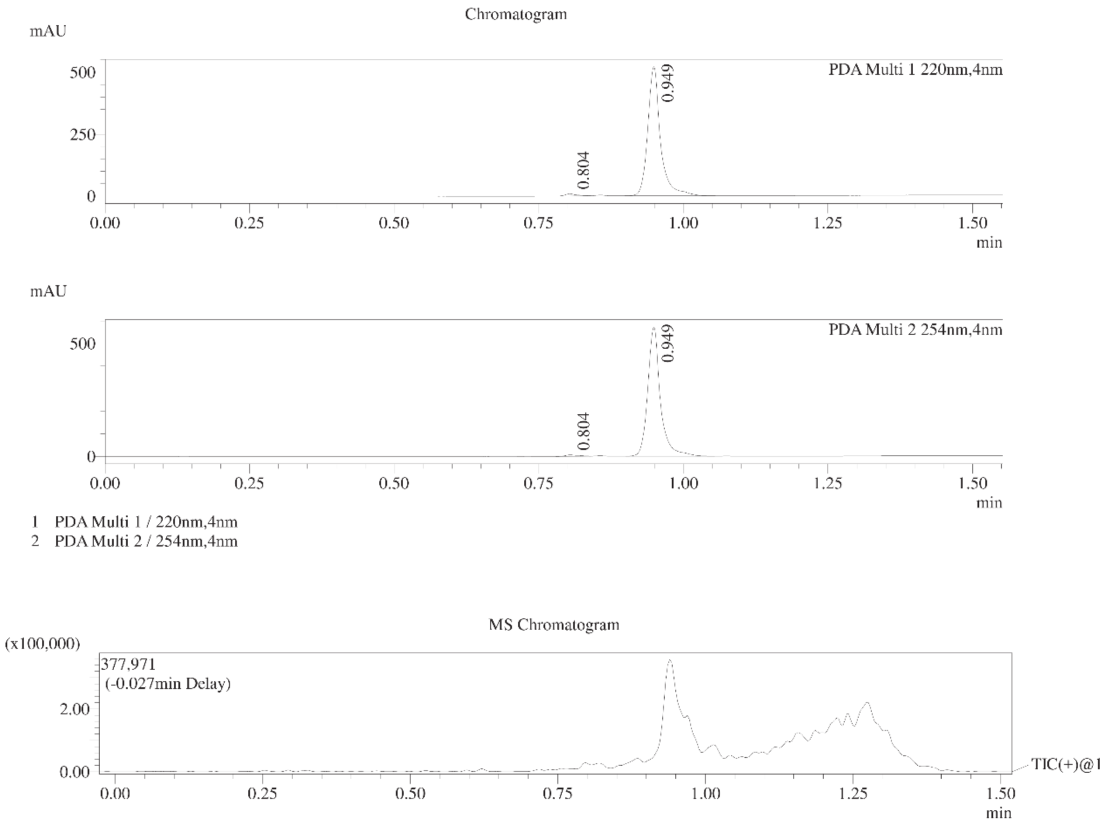

Integration Result

Peak Table

PDA Ch1 220nm

Peak#	Ret. Time	Height	Height%	USP Width	Area	Area%
1	0.804	8971	1.693	0.034	12182	1.492
2	0.949	520943	98.307	0.038	804502	98.508

Peak Table

PDA Ch2 254nm

Peak#	Ret. Time	Height	Height%	USP Width	Area	Area%
1	0.804	6669	1.152	0.041	10771	1.216
2	0.949	572454	98.848	0.038	875187	98.784

FIGURE S3 LC-MS was used to detect and characterize the Cpd16 and a summary of results.

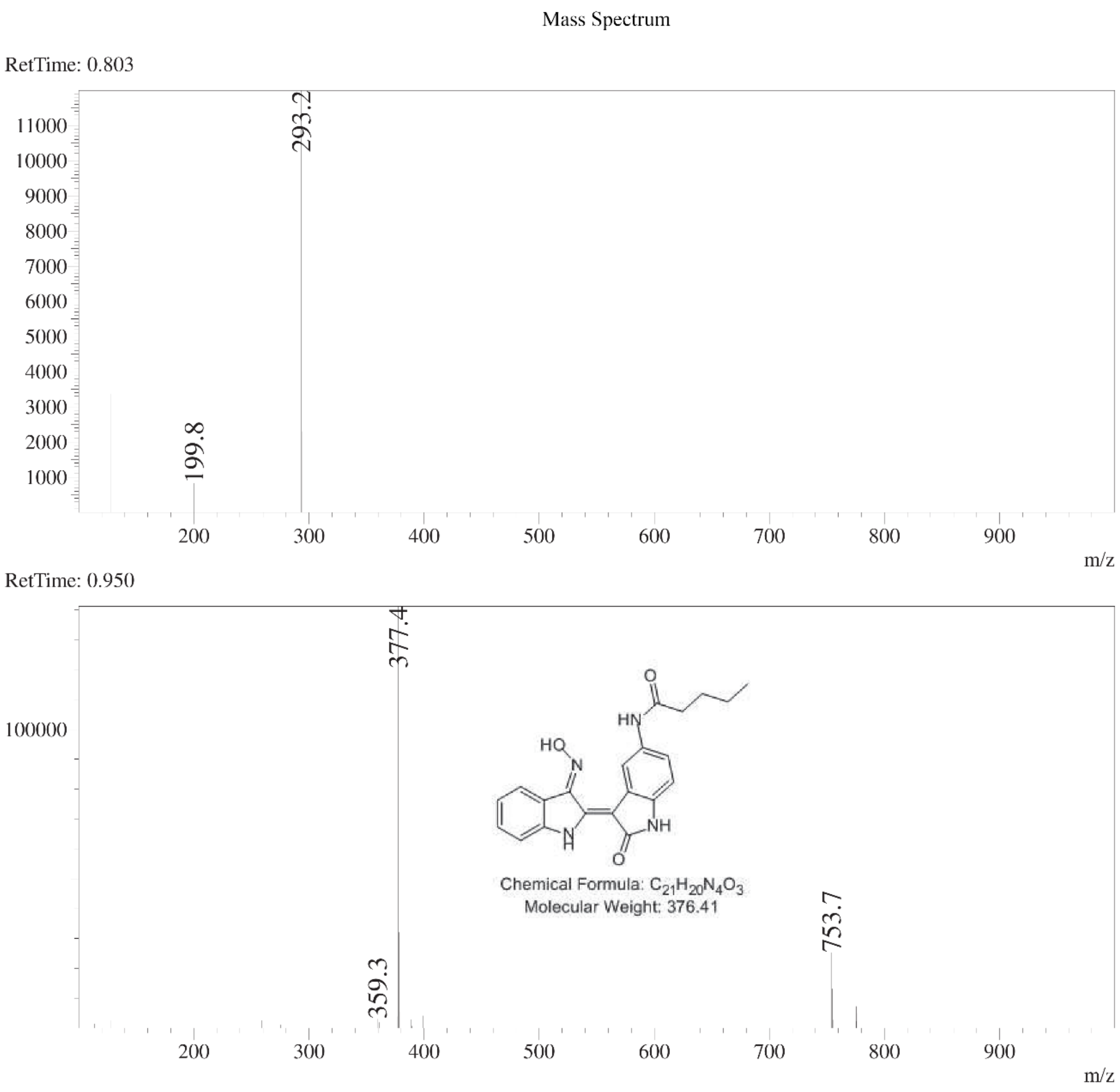

FIGURE S4 LC-MS report.

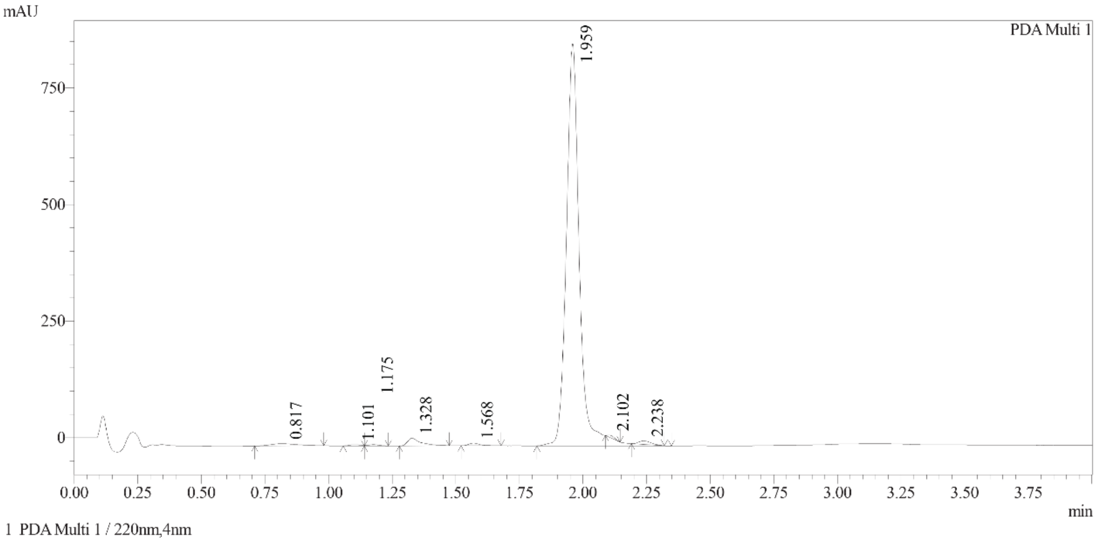

Integration result

PeakTable

PDA Ch1 220nm

Peak#	Ret. Time	USP Width	Resolution	Height	Area	Area %
1	0.817	0.198	0.000	4764	35569	1.094
2	1.101	0.083	2.024	2503	7276	0.224
3	1.175	0.078	0.927	2742	7725	0.238
4	1.328	0.081	1.920	16204	59037	1.816
5	1.568	0.077	3.054	5474	16663	0.513
6	1.959	0.087	4.765	863068	3088722	95.024
7	2.102	0.050	2.080	4013	9019	0.277
8	2.238	0.093	1.906	7758	26465	0.814
Total				906526	3250475	100.000

FIGURE S5 HPLC report at 220 nm and a summary of results.

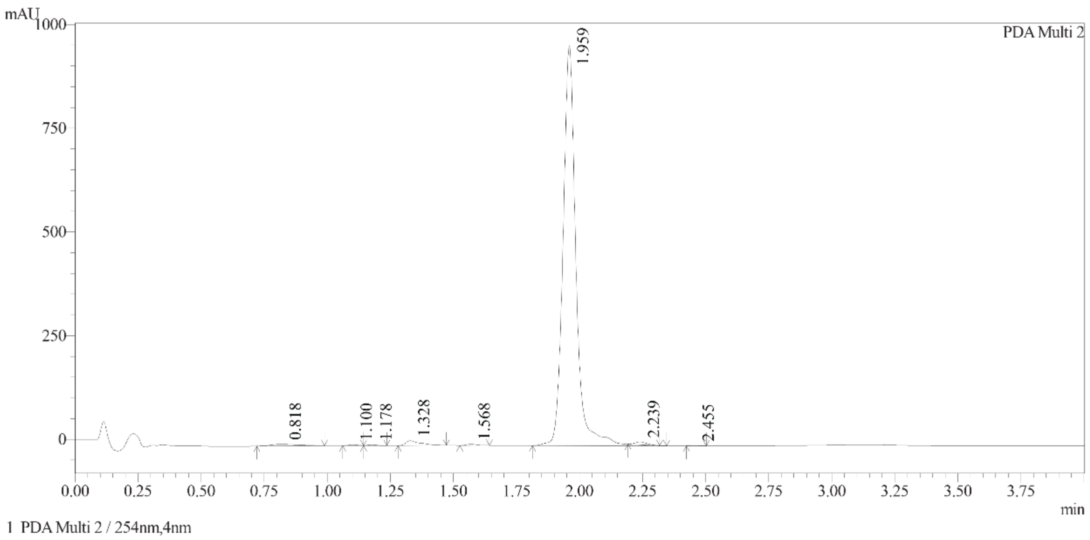

Integration result

PeakTable

PDA Ch2 254nm

Peak#	Ret. Time	USP Width	Resolution	Height	Area	Area %
1	0.818	0.199	0.000	4304	32008	0.900
2	1.100	0.077	2.038	2107	5901	0.166
3	1.178	0.069	1.069	2311	5948	0.167
4	1.328	0.090	1.901	11632	48398	1.361
5	1.568	0.074	2.923	3251	9098	0.256
6	1.959	0.087	4.834	963548	3428963	96.447
7	2.239	0.092	3.123	6838	23155	0.651
8	2.455	0.066	2.742	783	1826	0.051
Total				994774	3555298	100.000

FIGURE S6 HPLC report at 254 nm and a summary of results.

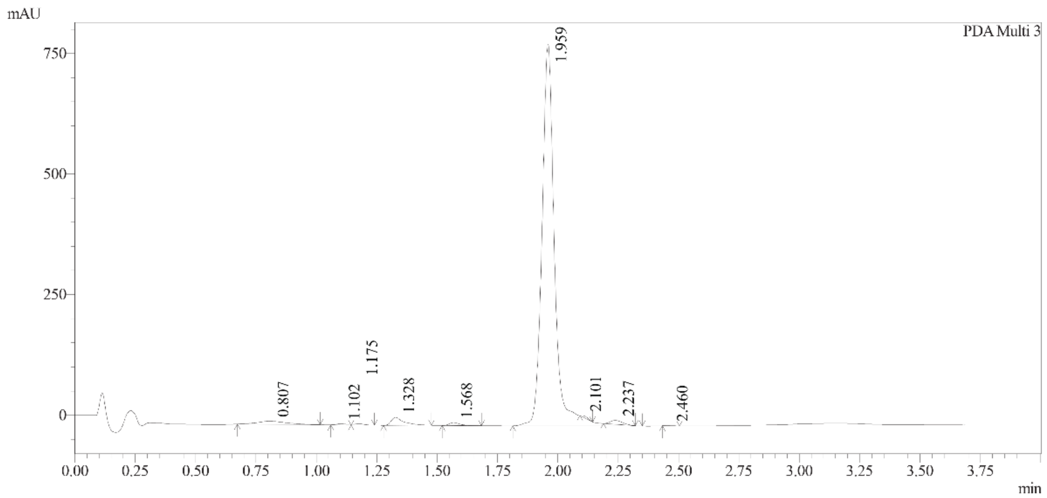

1 PDA Multi 3 / 215nm,4nm

Integration result

PeakTable

PDA Ch3 215nm

Peak#	Ret. Time	USP Width	Resolution	Height	Area	Area %
1	0.807	0.225	0.000	6454	55088	1.810
2	1.102	0.083	1.919	2340	6977	0.229
3	1.175	0.078	0.910	2837	7875	0.259
4	1.328	0.081	1.924	16395	59540	1.956
5	1.568	0.077	3.041	6090	18690	0.614
6	1.959	0.087	4.752	791902	2858950	93.921
7	2.101	0.045	2.159	2503	5708	0.188
8	2.237	0.097	1.924	8403	29741	0.977
9	2.460	0.059	2.864	672	1415	0.046
Total				837595	3043986	100.000

FIGURE S7 HPLC report at 215 nm and a summary of results.
